# Association of complement receptor 1 gene polymorphisms with cognitive function

**DOI:** 10.1152/physiolgenomics.00102.2017

**Published:** 2017-12-06

**Authors:** L. E. Zijlstra, J. W. Jukema, S. P. Mooijaart, M. A. de Vries, D. J. Stott, M. Castro Cabezas, S. Trompet

**Affiliations:** ^1^Department of Cardiology, Leiden University Medical Center, Leiden, The Netherlands; ^2^Department of Gerontology and Geriatrics, Leiden University Medical Center, Leiden, The Netherlands; ^3^Department of Internal Medicine, Center for Diabetes and Vascular Medicine, Franciscus Gasthuis, Rotterdam, The Netherlands; ^4^Department of Geriatric Medicine, University of Glasgow, Glasgow, Scotland

**Keywords:** Alzheimer’s disease, chromosome 1, cognitive function, complement receptor 1, older patients

## Abstract

Previous evidence suggest involvement of the complement receptor 1 (CR1) in development of Alzheimer’s disease. We investigated the association of CR1 gene polymorphisms with cognitive function in older subjects. Single nucleotide polymorphisms (SNPs) within the CR1 region on chromosome 1 (*n* = 73) were assessed in 5,244 participants in the PROspective Study of Pravastatin in the Elderly at Risk (51.9% female, mean age 75.3 yr). Linear regression, adjusted for age, sex, country, and use of pravastatin, was used to assess the association between the SNPs and cognitive function. All 73 SNPs within the genomic region of the CR1 gene on chromosome 1 were extracted. Eighteen were independent, according to a relatively stringent R^2^ threshold of >0.8 with LDlink. Twelve of the 18 investigated CR1 SNPs were significantly associated with a decline in cognitive function (all *P* < 0.05). These data indicate that genetic variation within the CR1 gene is associated not only with Alzheimer’s disease, but also with general cognitive function during late life.

## BACKGROUND/MOTIVATION FOR THE STUDY

Previous evidence suggest that the complement receptor 1 (CR1) may be involved in the development of multiple diseases. For instance, as previously reported, genetic variation within the CR1 gene is associated with inflammation and the risk of incident coronary artery disease (5). Also, in multiple genome-wide association studies (GWAS) concerning Alzheimer’s disease (AD) the CR1 locus has been identified as a candidate gene (3).

CR1 is a receptor for the complement proteins C3b and C4b. The complement system is involved in the innate immune system and functions as a first-line defense mechanism against infection. The complement system can be activated via different pathways, ultimately leading to activation of the protein C3, which is split into C3b and C3a. This leads to the facilitation of opsonization and phagocytosis of pathogens. Via the CR1 receptor, circulating erythrocytes can carry immune complexes and opsonized microorganisms to these phagocytic cells.

As described in a review by Alexander et al. (1) previous studies have described the complement system as not only involved in regulation of the innate immunity but also playing a role in the nervous system and therefore neurological disorders. As mentioned above, the CR1 gene has been associated to AD, a neurogenerative disease that is the most common cause of dementia in older patients. There is also evidence that variation within the CR1 gene is associated with general loss of cognitive function, which is the first clinical presentation of AD. For instance, Chibnik et al. (2) found an association between variation in CR1 (rs6656401) with cognitive aging in the general population.

Therefore, we investigated the association of CR1 gene polymorphisms with cognitive function in older patients.

## PHENOTYPE

We investigated the association of CR1 gene polymorphisms with cognitive function decline. A detailed description of how cognitive function was measured has been published previously (4).

At baseline cognitive dysfunction was screened with the Mini-Mental State Examination (MMSE). We used the generally accepted cut-off point of 24 (of 30) points. Participants with a score below this cut-off at baseline were excluded from PROspective Study of Pravastatin in the Elderly at Risk (PROSPER).

Outcome variables were derived from three other widely used cognitive function tests.

First, selective attention was assessed by the Stroop Color Word Test, which consists of three parts, namely color names, colored patches, and color names printed in incongruously colored ink. The time required to read the names or to identify colors is recorded. We used an abbreviated version of the test with 40 elements.

Second, the speed of processing of general information was assessed with the Letter-Digit Coding Test, which is a modification of the procedurally identical Symbol-Digits Modalities Test.

Third, verbal learning was assessed using the Picture-Word Learning Test, which is derived from the Groningen Fifteen Words Test. Outcomes are measured in three different trials and divided in recall and delayed recall after 20 min.

### 

#### Cohort details.

All data come from PROSPER, a study that investigated the relationship between statin treatment and the risk of coronary disease (*n* = 5,804). Measurement of cognitive decline was a prespecified end point. In summary, older participants were enrolled in Ireland, Scotland, and The Netherlands. Patients were included if they had a history of, or an increased risk for, vascular disease and a baseline cholesterol between 4.0 and 9.0 mmol/l. Of the 5,804 participants, 5,244 subjects were included for genetic analysis and 51.9% was female with a mean age of 75.3 ± 3.4 yr. Mean follow-up was 3.2 yr. As mentioned above, one of the exclusion criteria was poor cognitive function at baseline (MMSE <24). Detailed description, including all other in- and exclusion criteria, has been published previously (4).

#### Type of study.

Candidate gene.

#### Details of the SNPs studied.

From the GWAS, performed in PROSPER, all 73 SNPs within the genomic region of the CR1 gene on chromosome 1 were selected with PLINK software. According to LDlink, 18 SNPs were independent, taking a relatively stringent R^2^ threshold of >0.8. Details have been described previously.

A list of all 18 SNPs and additional references are included in the data supplement. (The online version of this article contains supplemental material.)

#### Analysis model.

Allele frequencies were estimated, and pairwise linkage disequilibrium (LD) between the investigated SNPs was estimated and plotted with the program Haploview. Associations between the CR1 SNPs and cognitive function were assessed with linear regression adjusted for sex, age, country, and treatment with pravastatin. PLINK statistical software was used for performance of all statistical analyses.

## RESULTS

All SNPs were in Hardy Weinberg equilibrium (*P* > 0.05). The results of the association between the 18 CR1 SNPs and four cognitive performance tests showed significant associations for 12 out of the 18 SNPs (all *P* < 0.05). A negative association with the Stroop Color Word Test indicates an increase in cognitive function, which is in line with a positive association for the other three tests. For nine SNPs the minor allele frequency was associated with an increase in cognitive function, whereas for three SNPs the minor allele frequency was associated with a decrease. Furthermore, four SNPs (rs9429944, rs4844599, rs11117956, rs12734030) showed statistical significant association with all four cognitive performance tests. Details of these SNPs, including scores for all four tests, are described in the results table in the online supplement.

## INTERPRETATION

Twelve of the 18 investigated CR1 SNPs were significantly associated with cognitive function. Therefore, these data indicate that genetic variation within the CR1 gene is not only associated with Alzheimer’s disease, but also with general cognitive function decline during late life. Furthermore, three of these 12 SNPs that were associated with improved cognitive function were associated with lower levels of CRP (5). These data further strengthen previous evidence of Alexander et al. (1) for the role of the complement system in cognitive function. However, future studies investigating the functionality of these CR1 SNPs are necessary.

## GRANTS

The PROSPER study was supported by an investigator-initiated grant obtained from Bristol-Myers Squibb. Prof. Dr. J. W. Jukema is an Established Clinical Investigator of the Netherlands Heart Foundation (Grant 2001 D 032). Support for genotyping was provided by the seventh framework program of the European commission (Grant 223004) and by the Netherlands Genomics Initiative (Netherlands Consortium for Healthy Aging Grant 050-060-810).

## DISCLOSURES

No conflicts of interest, financial or otherwise, are declared by the authors.

## AUTHOR CONTRIBUTIONS

L.E.Z., J.W.J., and S.T. interpreted results of experiments; L.E.Z. and S.T. drafted manuscript; L.E.Z., J.W.J., S.P.M., M.A.d.V., D.J.S., M.C.C., and S.T. edited and revised manuscript; L.E.Z., J.W.J., S.P.M., M.A.d.V., D.J.S., M.C.C., and S.T. approved final version of manuscript; J.W.J. and S.T. conceived and designed research; J.W.J. and S.T. analyzed data; S.T. performed experiments; S.T. prepared figures.

## Supplemental Data

Supplemental Data Supplemental Data - .pdf (487 KB)

## References

[1] Alexander JJ, Anderson AJ, Barnum SR, Stevens B, Tenner AJ The complement cascade: Yin-Yang in neuroinflammation–neuro-protection and -degeneration. J Neurochem 107: 1169–1187, 2008. doi:10.1111/j.1471-4159.2008.05668.x. 18786171 PMC4038542

[2] Chibnik LB, Shulman JM, Leurgans SE, Schneider JA, Wilson RS, Tran D, Aubin C, Buchman AS, Heward CB, Myers AJ, Hardy JA, Huentelman MJ, Corneveaux JJ, Reiman EM, Evans DA, Bennett DA, De Jager PL CR1 is associated with amyloid plaque burden and age-related cognitive decline. Ann Neurol 69: 560–569, 2011. doi:10.1002/ana.22277. 21391232 PMC3066288

[3] Fonseca MI, Chu S, Pierce AL, Brubaker WD, Hauhart RE, Mastroeni D, Clarke EV, Rogers J, Atkinson JP, Tenner AJ Analysis of the putative role of CR1 in Alzheimer’s disease: genetic association, expression and function. PLoS One 11: e0149792, 2016. doi:10.1371/journal.pone.0149792. 26914463 PMC4767815

[4] Shepherd J, Blauw GJ, Murphy MB, Cobbe SM, Bollen EL, Buckley BM, Ford I, Jukema JW, Hyland M, Gaw A, Lagaay AM, Perry IJ, Macfarlane PW, Meinders AE, Sweeney BJ, Packard CJ, Westendorp RG, Twomey C, Stott DJ The design of a prospective study of Pravastatin in the Elderly at Risk (PROSPER). PROSPER Study Group. PROspective Study of Pravastatin in the Elderly at Risk. Am J Cardiol 84: 1192–1197, 1999. doi:10.1016/S0002-9149(99)00533-0. 10569329

[5] de Vries MA, Trompet S, Mooijaart SP, Smit RA, Böhringer S, Castro Cabezas M, Jukema JW Complement receptor 1 gene polymorphisms are associated with cardiovascular risk. Atherosclerosis 257: 16–21, 2017. doi:10.1016/j.atherosclerosis.2016.12.017. 28033544 PMC7094315

