# Supplementary material for: Association of complement receptor 1 gene polymorphisms with cognitive function
Source: Physiol Genomics. 2017 Dec 6;50(2):102–3. doi: 10.1152/physiolgenomics.00102.2017 (PMC5867616; doi:10.1152/physiolgenomics.00102.2017)
Supplement: Supplemental Data — pdf (487 KB) [file supplemental-data.pdf]

References

1. Alexander JJ, Anderson AJ, Barnum SR, Stevens B, Tenner AJ. The complement cascade: Yin-Yang in neuroinflammation--neuro-protection and -degeneration. Journal of neurochemistry. 2008;107(5):1169-87.

2. Brand N, Jolles J. Learning and retrieval rate of words presented auditorily and visually. J Gen Psychol 1985;112:201–210.

3. Carrasquillo MM, Belbin O, Hunter TA, Ma L, Bisceglio GD, Zou F, et al. Replication of CLU, CR1, and PICALM associations with alzheimer disease. Archives of neurology. 2010;67(8):961-4.

4. Chibnik LB, Shulman JM, Leurgans SE, Schneider JA, Wilson RS, Tran D, et al. CR1 is associated with amyloid plaque burden and age-related cognitive decline. Annals of neurology. 2011;69(3):560-9.

5. Corneveaux JJ, Myers AJ, Allen AN, Pruzin JJ, Ramirez M, Engel A, et al. Association of CR1, CLU and PICALM with Alzheimer's disease in a cohort of clinically characterized and neuropathologically verified individuals. Human molecular genetics. 2010;19(16):3295-301.

6. Deelman BG, Brouwer WH, Zomeren AHv, Saan RJ. Functiestoornissen na trauma capitis. In: A Jennekens-Schinkel, JJ Diamant, HFA Diesfeldt, R Haaxma, eds. Neuropsychologie in Nederland Deventer. The Netherlands: Van Loghum Slaterus, 1980:253–281.

7. Fearon DT. Identification of the membrane glycoprotein that is the C3b receptor of the human erythrocyte, polymorphonuclear leukocyte, B lymphocyte, and monocyte. The Journal of experimental medicine. 1980;152(1):20-30.

8. Folstein MF, Folstein SE, McHugh PR. Mini-mental state: a practical method for grading the cognitive state of patients for the clinician. J Psychiatr Res 1975;12:189 –198.

9. Fonseca MI, Chu S, Pierce AL, Brubaker WD, Hauhart RE, Mastroeni D, et al. Analysis of the Putative Role of CR1 in Alzheimer's Disease: Genetic Association, Expression and Function. PloS one. 2016;11(2):e0149792.

10. Houx PJ, Jolles J, Vreiling FW. Stroop interference: aging effects assessed with the Stroop Color-Word Test. Experiment Aging Res 1993;19:209 –224.

11. Houx PJ, Shepherd J, Blauw GJ, Murphy MB, Ford I, Bollen EL, et al. Testing cognitive function in elderly populations: the PROSPER study. PROspective Study of Pravastatin in the Elderly at Risk. Journal of neurology, neurosurgery, and psychiatry. 2002;73(4):385-9.

12. Jun G, Naj AC, Beecham GW, Wang LS, Buros J, Gallins PJ, et al. Meta-analysis confirms CR1, CLU, and PICALM as alzheimer disease risk loci and reveals interactions with APOE genotypes. Archives of neurology. 2010;67(12):1473-84.

13. Kisserli A, Tabary T, Cohen JHM, Duret V, Mahmoudi R. High-resolution Melting PCR for Complement Receptor 1 Length Polymorphism Genotyping: An Innovative Tool for Alzheimer's Disease Gene Susceptibility Assessment. Journal of visualized experiments : JoVE. 2017(125).

14. Klein M, Ponds RWHM, Houx PJ, Jolles J. Effect of test duration on age related differences in Stroop interference. J Clin Exptl Neuropsychol 1997;1:66–81.

15. Lambert JC, Heath S, Even G, Campion D, Sleegers K, Hiltunen M, et al. Genome-wide association study identifies variants at CLU and CR1 associated with Alzheimer's disease. Nature genetics. 2009;41(10):1094-9.

16. Machiela MJ, Chanock SJ. LDlink: a web-based application for exploring population-specific haplotype structure and linking correlated alleles of possible functional variants, Bioinformatics 31 (2015) 3555e3557.

17. Lezak MD. Verbal functions and language skills. In: Lezak MD, ed. Neuropsychological Assessment. 2nd ed. New York: Oxford University Press, 1995, 554–555.

18. Postmus I, Trompet S, de Craen AJ, Buckley BM, Ford I, Stott DJ, et al. PCSK9 SNP rs11591147 is associated with low cholesterol levels but not with cognitive performance or noncardiovascular clinical events in an elderly population. J Lipid Res. 2013; 54(2): 561-566.

19. Rey A. L'examen psychologique dans les cas d'encephalopathie traumatique. Paris, France: Presses Universitaires de France, 1964

20. Ricklin D, Lambris JD. Complement in immune and inflammatory disorders: pathophysiological mechanisms. Journal of immunology (Baltimore, Md : 1950). 2013;190(8):3831-8.

21. Shepherd J, Blauw GJ, Murphy MB, Cobbe SM, Bollen EL, Buckley BM, et al. The design of a prospective study of Pravastatin in the Elderly at Risk (PROSPER). PROSPER Study Group. PROspective Study of Pravastatin in the Elderly at Risk. The American journal of cardiology. 1999;84(10):1192-7.

22. Shiffman D, Trompet S, Louie JZ, Rowland CM, Catanese JJ, Iakoubova OA, et al. Genome-wide study of gene variants associated with differential cardiovascular event reduction by pravastatin therapy. PloS one. 2012;7(5):e38240.

23. Sjoberg AP, Trouw LA, Blom AM. Complement activation and inhibition: a delicate balance. Trends in immunology. 2009;30(2):83-90.

24. Smith A. The Symbol Digit Modalities Test. A neuropsychologic test for economic screening of learning and other cerebral disorders. Learning Disorders 1968;3:82–91.

25. Trompet S, Craen de AJ, Postmus I, Ford I, Sattar N, Caslake M, Stott DJ, et al. Replication of LDL GWAs hits in PROSPER/PHASE as validation for future (pharmaco)genetic analyses. BMC medical genetics. 2011; 12: 131.

26. Trompet S, van Vliet P, de Craen AJ, Jolles J, Buckley BM, Murphy MB, et al. Pravastatin and cognitive function in the elderly. Results of the PROSPER study. J Neurol. 2010; 257(1): 85-90.

27. de Vries MA, Trompet S, Mooijaart SP, Smit RA, Bohringer S, Castro Cabezas M, et al. Complement receptor 1 gene polymorphisms are associated with cardiovascular risk. Atherosclerosis. 2017;257:16-21.

rsID	Locus name	Chr	Position	EA/ALT	Trait	EAF
rs9429944	CR1	1	205736930	T/A	Stroop	0.2342
					LDT	
					PLTi	
					PLTd	
rs11117956	CR1	1	205750982	G/T	Stroop	0.1548
					LDT	
					PLTi	
					PLTd	
rs3886100	CR1	1	205805750	A/G	Stroop	0.3582
					LDT	
					PLTi	
					PLTd	
rs4844609	CR1	1	205849539	A/T	Stroop	0.009695
					LDT	
					PLTi	
					PLTd	
rs12734030	CR1	1	205860587	T/C	Stroop	0.1318
					LDT	
					PLTi	
					PLTd	

Abbreviations: rsID - dbSNP rsID; locus name, variant annotation; Chr - chromosome;
EAF - Effect allele frequency; β (SE)/Z-score - effect estimate and standard error for a
for association; N - number of samples analysed;

β (SE)/Z-score	*P*	N
-1,63(0,638)/-2,554	0.01067	4822
0,5938(0,1833)/3,239	0.001206	
0,08935(0,04249)/2,103	0.03553	
0,1324(0,06159)/2,149	0.03166	
-2,365(0,7877)/-3,002	0.002695	4219
0,9442(0,2277)/4,146	3.44E-05	
0,1819(0,05319)/3,421	0.0006308	
0,2474(0,07693)/3,216	0.001308	
-1,717(0,639)/-2,687	0.007235	3679
0,7256(0,1848)/3,926	8.81E-05	
0,1286(0,04266)/3,014	0.002595	
0,09809(0,06189)/1,585	0.1131	
-8,286(2,868)/-2,89	0.003876	4475
3,406(0,8619)/3,952	7.88E-05	
0,04887(0,1929)/0,2533	0.8001	
-0,0762(0,278)/-0,2741	0.784	
-2,398(0,8084)/-2,966	0.003027	4752
0,8161(0,2319)/3,519	0.0004376	
0,1245(0,05413)/2,3	0.02148	
0,2316(0,07839)/2,955	0.003148	

Pos - Build 37 position; EA/ALT - Effect allele and alternative allele; Trait;
a quantitative trait and Z-score for example for a binary trait; *P* - *P* -value

Study genotyping / QC

Cohort Name	Array used and version	Genotype calling software	Related individuals (yes/no)?	Familial adjustment method (if applicable)	Population stratification assessment and adjustment
PROSPER	Illumina 660K Beadchip	Beadstudio	no	exclusion based on IBS clustering, checked for duplicates. First and second degree relatives	exclusion based on IBS clustering

	SNP QC					
**Analysis software version**	**MAF**	**Call Rate**	**HWE p-value**	**# SNPs analysed post-QC**	**Other filtering**	**Number of SNPs used for imputation**
PLINK version 1.07	NA	>97.5%	>10-6	557,192	No	557,192

Imputation information					Sample QC
**Imputation software**	**Imputation backbone, if 1000 genomes indicate the release**	**Haplotypes used for backbone**	**NCBI build**	**Chr X imputed yes/no**	**Call Rate**
MACH	HapMAP reference panel 36	NA	36.2	No	>95%

Other QC exclusions (e.g. IBS clustering, heterozygosity, other)
Duplicate and MZ samples, sex mismatch, non-caucasians

Supplement – list of SNPs

1. rs9429944
2. rs1831150
3. rs1986158
4. rs4844597
5. rs1887632
6. rs9429945
7. rs4844599
8. rs11117956
9. rs6656401
10. rs12729446
11. rs3886100
12. rs12038575
13. rs10127904
14. rs12144461
15. rs17259045
16. rs4844609
17. rs12734030
18. rs6661764
